# A Dying Cell's Last Hurrah: Flagging the Remnants of a Fatal Infection

**DOI:** 10.1371/journal.pbio.0030197

**Published:** 2005-04-26

For a cell, suicide is a perfectly acceptable response to stress. Programmed cell death, or apoptosis, is a normal part of development: it occurs 131 times over the course of worm development, for example, and throughout the life of an organism to replenish worn out cells in a variety of tissues. Disease and infection also trigger apoptosis. The dying cell shrinks into a membrane-bound package that attracts the attention of phagocytes, which quickly ingest and degrade the cell's contents. Dendritic cells, specialized phagocytes, patrol the body, capturing molecules (or antigens) expressed by a host of pathogens, parasites, environmental substances, and apoptotic cells, and present their findings to the helper and killer T cells that launch an immune response.

Before dendritic cells can sound the immune alarm, they must capture antigens, chop them into peptide fragments, and bind the antigenic peptides to molecules of the major histocompatibility complex (MHC). Though dendritic cells can present to helper or killer T cells, it had been thought that the MHC I–bound antigens (recognized by killer T cells) must first enter the dendritic cell's cytosol and undergo proteasome degradation. It was also thought that antigenic peptides needed the transporter activity of TAP (transporter associated with antigen processing) proteins to escort them into the cellular compartment where the MHC I molecules reside.

A new study by Nathalie Blachère, Robert Darnell, and Matthew Albert lends support to an emerging view that antigen processing may be more flexible and suggests that dendritic cells can generate MHC I peptides that activate killer T cells through cross-presentation. (The phenomenon is called cross-presentation because the pathway crosses established rules of MHC I antigen presentation.) Working with a mouse model to study killer T cell activation in response to influenza antigens derived from apoptotic cells, the authors show that two distinct pathways mediate antigen processing. In the first pathway, antigen processing in dendritic cells depends on TAP, following the established model. But in the second pathway, antigens processed by a dying, infected cell can bypass the dendritic cells' degradation machinery yet still permit generation of MHC I/peptide complexes that can engage killer T cells.

T cells can recognize millions of different antigens because each T cell is outfitted with a unique T cell antigen receptor that recognizes a unique MHC/peptide complex. In this study, the authors used the activation of influenza-reactive T cells as evidence of antigen presentation. To test the hypothesis that dying cells can process antigen for cross-presentation, Blachère et al. limited the dendritic cells' ability to process antigen by using cells extracted from the bone marrow of mice lacking functional TAP proteins. If the cells could provoke a T cell response, then antigen-specific MHC I/peptide complexes would have to arise through an alternate pathway.

When TAP-deficient dendritic cells were directly infected with influenza, they could not elicit a T cell response. But when infected dying cells were internalized by the TAP-deficient dendritic cells, the dendritic cells were able to cross-present influenza antigen and activate the T cells. To investigate the mechanism of cross-presentation, the authors generated cells with deficient proteasomes that could not degrade viral proteins, and also used another cell line inhibited in TAP activity. When either of these cell types were infected with influenza, caused to undergo apoptosis, and engulfed by dendritic cells, it became clear that the dendritic cells needed functional TAP to successfully present the influenza antigen to T cells. The authors then went on to validate the operation of these pathways in vivo.[Fig pbio-0030197-g001]

**Figure pbio-0030197-g001:**
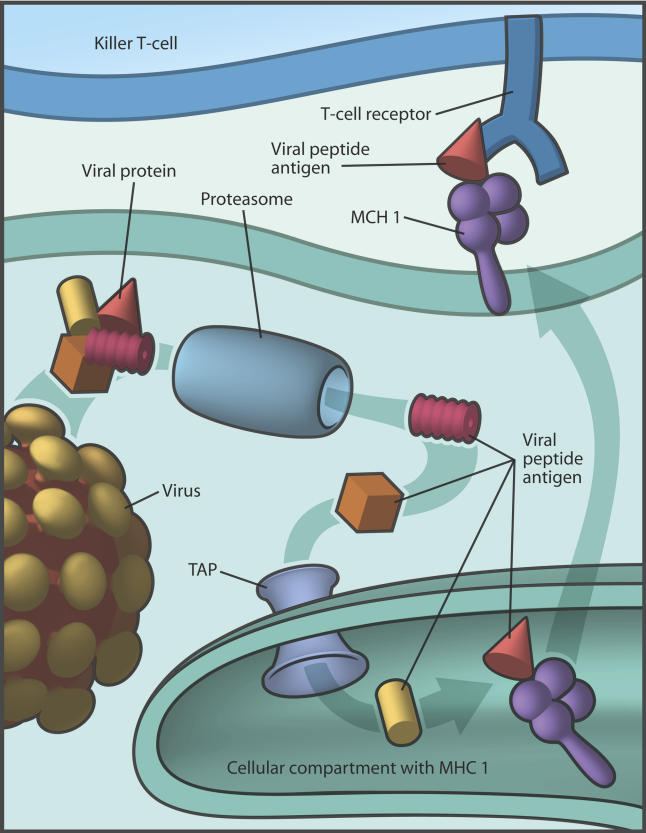
Antigenic proteins in the cytosol are degraded by the proteasome and transported by TAP into the MHC1 compartment for MHC1 loading (Image: Giovanni Maki)

Altogether, these results point to two MHC I pathways: one relies on transporter activity in the dendritic cell; the other allows dendritic cells to capture antigens already processed in the dying cell. Though the details of the cross-presentation pathway remain unclear, the conditions that give rise to it are not: when dendritic cells are directly infected, they mediate antigen processing. When dendritic cells ingest already infected, apoptotic cells, the dying cells deliver processed antigen and set the stage for cross-presentation. It's possible that stressful conditions, like an influenza infection, trigger this cross-presentation pathway, allowing the dying cell to alert the immune system to a pathogenic agent that requires immediate action.

